# Nurse-led cardiac rehabilitation improves quality of life in elderly CAD patients: A retrospective cohort study

**DOI:** 10.1097/MD.0000000000044939

**Published:** 2025-10-24

**Authors:** Juan Feng, Shasha Li, Jie Liu

**Affiliations:** a General Medicine Ward l, The Sixth Hospital of Wuhan, Affiliated Hospital of Jianghan University, Wuhan, Hubei Province, China.

**Keywords:** cardiac rehabilitation, coronary artery disease, elderly, nurse-led, propensity score matching, quality of life

## Abstract

To assess the effect of nurse-led cardiac rehabilitation programs on improving the quality of life of elderly patients with coronary artery disease (CAD). This single-center retrospective cohort study included elderly CAD patients hospitalized and followed at our hospital from June 2022 to June 2024. Based on receipt of a nurse-led rehabilitation program, patients were assigned to a rehabilitation or conventional group. Propensity score matching (1:1 nearest neighbor) was performed using variables including age, gender, major comorbidities (hypertension, diabetes, and hyperlipidemia), body mass index, smoking status, NYHA class, left ventricular ejection fraction, history of percutaneous coronary intervention/coronary artery bypass grafting, and baseline medication use. The program comprised individualized assessment and goal setting before discharge, plus remote support, and self-management training after discharge. Primary outcome was the SF-36 quality of life score; secondary outcomes were Generalized Anxiety Disorder-7, Patient Health Questionnaire-9, and Six-Minute Walk Test. Assessments were at discharge, 3 months, and 6 months. After matching, baseline characteristics were balanced between the 2 groups. There were no significant differences between the rehabilitation group and the conventional group in age (71.6 ± 6.4 vs 72.1 ± 6.1 years), gender (male 61.3% vs 64.5%), comorbidity of hypertension (72.6% vs 75.8%), and percutaneous coronary intervention ratio (50.0% vs 48.4%) (all *P* > .05). At 6 months of follow-up, the rehabilitation group showed a significant improvement in SF-36 total score (from 58.2 ± 7.4–75.6 ± 5.8), which was better than the conventional group (from 57.6 ± 6.9–65.2 ± 6.5) (interaction *P* = .023). The most significant improvements were observed in physical function and social function (interaction *P* = .017 and 0.026, respectively). The physical function score in the rehabilitation group at 6 months was 78.9 ± 6.2, significantly higher than the conventional group’s score of 68.5 ± 6.9. The Generalized Anxiety Disorder-7 score in the rehabilitation group decreased from a median of 7 at discharge to 3 at 6 months (IQR 2–5), while the conventional group remained at 6 (IQR 4–8), with a significant difference (*P* < .01). A similar trend was observed in Patient Health Questionnaire-9 scores. The 6-minute walking distance also showed greater improvement in the rehabilitation group (from 298.5 ± 45.2–385.7 ± 38.4 m, *P* < .001). Nurse-led cardiac rehabilitation significantly improves quality of life, reduces anxiety and depression, and enhances functional capacity in elderly CAD patients, supporting its broader clinical application.

## 1. Introduction

With the aging population becoming increasingly prominent in modern society, cardiovascular diseases, particularly coronary artery disease (CAD), have emerged as one of the major chronic conditions affecting the health and quality of life of elderly individuals.^[1,2]^ According to data released by the World Health Organization and the National Cardiovascular Disease Center, the prevalence of CAD among individuals aged 60 and above in China has been steadily increasing. Due to its chronic nature and recurrent episodes, CAD often coexists with psychological issues such as anxiety and depression, as well as physical decline and limited social functioning (SF), significantly affecting the patient’s quality of life and self-management abilities.^[3–5]^ Therefore, how to assist elderly CAD patients in achieving functional recovery, emotional adjustment, and the long-term adherence to healthy behaviors after the condition stabilizes has become a key issue in cardiovascular prevention and treatment strategies.

Cardiac rehabilitation (CR), as a comprehensive secondary prevention model, has been repeatedly emphasized in multiple international guidelines (e.g., AHA/ACC, ESC) over the past few decades and has been proven to significantly reduce cardiovascular event risk, improve quality of life, and reduce readmission rates.^[6,7]^ However, during the actual clinical promotion, particularly in developing regions such as China, elderly populations show generally low rehabilitation adherence, and the implementation and coverage rates of rehabilitation programs are far from meeting the demand.^[8]^ Studies have shown that traditional doctor-led rehabilitation models are often inflexible and discontinuous when addressing the diverse rehabilitation needs of elderly patients, due to issues such as centralized resources, fixed formats, and a lack of continuous follow-up.^[9]^

In this context, nurse-led CR models have gradually attracted widespread attention. As continuous caregivers in the rehabilitation process, specialized nurses have natural advantages and good accessibility in disease education, behavioral intervention, psychological counseling, and remote follow-up. In recent years, more studies have attempted to integrate the nurse-led concept into CR services, aiming to improve patient participation and rehabilitation outcomes through systematic health management and personalized support. This is especially important for elderly CAD patients who have declining cognitive abilities, emotional vulnerability, and high dependency, where the ongoing guidance and emotional companionship provided by nurses may play a crucial role in helping them establish stable and healthy behavior patterns.^[10,11]^

The nurse-led CR model provides a new approach to address the aforementioned issues. This model fully leverages the professional advantages of nursing staff in health management and differentiates itself from traditional models through the following features^[5,12]^: continuous care, extending from hospitalization to the community and home; individualized intervention, based on comprehensive assessments to formulate targeted plans; multidimensional support, defined in this study as a comprehensive intervention that includes physical rehabilitation (exercise training, dietary management, and cardiovascular risk factor control), psychological support (emotional counseling, anxiety/depression assessment, and coping skills training), and SF enhancement (family education, peer support, and facilitation of community reintegration). Particularly with the support of information technology, nurses can provide continuous professional guidance to patients through remote monitoring and regular follow-ups.

Existing evidence on nurse-led CR for elderly CAD patients remains limited, especially in China. Several studies abroad, such as Chiarini et al^[5]^ and Clark et al,^[12]^ have demonstrated that structured nurse-led interventions can significantly improve physical capacity and psychological well-being in elderly coronary patients. These programs often integrate individualized exercise training, psychosocial counseling, and long-term follow-up, yielding high adherence rates in developed healthcare systems. However, their models are typically resource-intensive and rely heavily on specialized rehabilitation centers, which may not be feasible in many developing regions.

In contrast, most domestic studies have focused on general patient education or short-term hospital-based rehabilitation, with limited incorporation of continuous remote support or behavior change theory. Few have adopted a comprehensive, multi-dimensional nurse-led model specifically tailored to elderly CAD patients, combining in-hospital assessment with post-discharge telehealth follow-up. Our study addresses this gap by implementing a structured program led by trained cardiovascular nurses, integrating personalized goal-setting, digital platform monitoring, and sustained self-management support. Compared with previous international models, our approach emphasizes scalability and adaptability to resource-limited settings, while maintaining multi-domain outcome evaluation (quality of life, psychological state, and functional status).

## 2. Materials and methods

### 2.1. Study design

This research was approved by the Ethics Committee of Affiliated Hospital of Jianghan University (Approval No. 2022-CR-015). This study is a retrospective cohort study, approved by the Ethics Committee of our hospital. The study subjects were elderly patients with CAD who were treated and followed up at our hospital between June 2022 and June 2024. Based on whether patients received a nurse-led CR program after discharge, they were divided into a rehabilitation program group and a conventional group. As this study was based on existing medical records and follow-up data, the requirement for written informed consent was waived by the Ethics Committee. The study was conducted in accordance with the Declaration of Helsinki and relevant national ethical guidelines.

Patients in the rehabilitation program group participated in a CR management program led by a professional nursing team after discharge, which included health education, exercise training guidance, psychological support, nutritional interventions, and regular follow-up. Patients in the conventional group received only standard discharge guidance and outpatient follow-up. To control for potential confounding factors, this study used propensity score matching (PSM) with a 1:1 nearest-neighbor matching method, matching variables such as age, gender, comorbidities, and disease severity. A total of 62 patients were included in each group.

### 2.2. Inclusion and exclusion criteria

*Inclusion criteria*: age ≥ 60 years; hospitalized and diagnosed with CAD at our hospital between June 2022 and June 2024, including stable angina, unstable angina, myocardial infarction, or patients post-percutaneous coronary intervention (PCI) or coronary artery bypass grafting; clear records of participation in the nurse-led CR program (including rehabilitation start time, content, frequency, etc), or receiving conventional follow-up; complete medical records, including admission records, diagnostic certificates, discharge summaries, and nursing records; complete follow-up data, including at least 6 months of quality of life assessment data post-discharge (e.g., SF-36 scale, World Health Organization Quality of Life-Brief scores, etc).

*Exclusion criteria*: severe liver or kidney dysfunction, or malignant tumors; mental or cognitive disorders affecting the quality of life evaluation (e.g., Alzheimer disease); incomplete data or missing quality of life assessments; death or loss to follow-up during the study period; previous participation in other interventional clinical trials.

### 2.3. Nursing plan

#### 2.3.1. Rehabilitation program group (nurse-led cardiac rehabilitation nursing group)

Patients in the rehabilitation program group received comprehensive, nurse-led rehabilitation services after discharge. The intervention was based on health behavior change theory, tailored to each patient’s condition, and dynamically managed through an information platform.

The nurse intervention team consisted of cardiovascular specialist nurses who met the following qualification standards: at least 5 years of clinical nursing experience in cardiology, certification in CR nursing, and formal training in psychological counseling skills. Before the study, all team members completed a standardized 20-hour training program covering CAD management guidelines, exercise prescription, dietary guidance, psychological assessment tools (Generalized Anxiety Disorder [GAD-7] and Patient Health Questionnaire [PHQ-9]), communication skills, and operation of the rehabilitation platform. Competency was assessed through a written test (passing score ≥ 85/100) and a practical skills evaluation, with only those meeting both standards authorized to conduct interventions.

##### 2.3.1.1. Hospital assessment and goal setting stage

During hospitalization, the responsible nurse conducted a systematic assessment, including CAD risk factors, self-management ability, psychological status, and exercise tolerance (e.g., Six-Minute Walking Test [6MWT]). Based on the results, the nurse worked with the patient to set individualized goals covering regular exercise, balanced diet, emotional regulation, smoking cessation, and daily healthy habits. Patients received a rehabilitation guidebook, along with an explanation of the follow-up process and online support system. Group education sessions (5–8 patients) were organized to improve health awareness and peer support. Patients were also invited to join an online rehabilitation group (e.g., WeChat) for ongoing communication, reminders, and experience sharing.

##### 2.3.1.2. Post-discharge remote support and self-management stage

After discharge, patients reported daily health metrics on the rehabilitation platform, such as steps, exercise frequency and duration, dietary adherence, emotional status, and smoking status. The platform provided graphical feedback with color-coded prompts (red–yellow–green) and offered multimedia learning resources, including case videos, dietary demonstrations, and emotional management courses. Nurses reviewed data trends weekly, provided timely feedback, identified issues, and adjusted goals as needed.

During the study period, patients in the rehabilitation group logged into the platform an average of 4.2 ± 1.1 times per week, with a weekly health metric submission compliance rate of 86.5%. Response rates to nurse feedback messages exceeded 90%, and >75% of patients actively participated in at least one group discussion per month. Patients could post questions on the platform, which nurses or peers would answer to encourage knowledge sharing and emotional support. The overall aim was to enhance patients’ self-efficacy and sustain healthy behaviors through gradual goal setting and continuous interaction, thereby improving their quality of life.

### 2.4. Conventional group (control group)

Patients in the conventional group received brief health education from the inpatient nurse before discharge. The content included: oral explanation of basic CAD knowledge, medication instructions, brief guidance on exercise and dietary principles (e.g., low-salt, low-fat diet, moderate aerobic exercise), and smoking cessation advice for smokers. They were given a paper health education manual.

This group did not receive personalized goal setting, remote monitoring, or systematic follow-up interventions. During data collection, researchers taught patients how to use pedometers to assist with exercise monitoring, but these patients were not included in the rehabilitation guidance system. Throughout the study period, patients in this group were not included in the online communication platform or rehabilitation support program.

### 2.5. Data collection

This study collected data retrospectively from elderly patients diagnosed with CAD and who received standard follow-up care at our hospital between June 2022 and June 2024. Patient medical records were retrieved from the hospital’s electronic health information system, covering basic demographic characteristics (e.g., age, gender, education level, smoking history, and body mass index), clinical data (including comorbidities such as hypertension, diabetes, hyperlipidemia, heart function classification, and length of hospital stay), treatment methods (e.g., whether they underwent PCI or coronary artery bypass grafting, medication usage), and CR participation (including content, start time, frequency, etc). The patients were grouped into the intervention group (nurse-led CR program) and the control group based on whether they received the nurse-led rehabilitation program after discharge. The researchers reviewed discharge guidance records, rehabilitation follow-up records, and platform interaction data to verify the implementation of the rehabilitation intervention, assessing the intervention’s effectiveness based on its completeness, continuity, and adherence.

Quality of life data were collected using standardized tools, primarily using the Chinese version of the SF-36 scale. This scale assesses 8 dimensions: physical functioning (PF), role-physical, SF, vitality, mental health, bodily pain, general health, and role-emotional. The total score and dimension scores were included in the analysis. Patients were required to complete the scale assessments at discharge, and 3 and 6 months post-discharge. Data were collected by follow-up nurses, who were trained uniformly to complete phone follow-ups or outpatient visits and enter the data into the database. For psychological health, the GAD-7 and PHQ-9 were used to assess psychological status, with data collected at the same 3 follow-up time points to ensure independence and authenticity of responses. Additionally, the study collected 6MWT results related to functional status, which were conducted by CR nurses under standardized conditions to ensure data consistency and comparability. Cardiovascular risk factor data, such as blood pressure and blood lipid levels, were obtained through outpatient follow-up or community monitoring. Focused data included systolic and diastolic blood pressure, total cholesterol, and low-density lipoprotein cholesterol at 3 and 6 months post-discharge. Data sources included outpatient laboratory systems, home monitoring records (with photo verification), and nursing follow-up manuals.

All data were independently entered by 2 researchers and cross-checked before being stored in an encrypted electronic database to ensure data completeness and accuracy. In case of missing data, the research team made efforts to complete the data by revisiting patients, reviewing medical records, or contacting patients and their families. Core variable data or missing follow-up data that could not be retrieved were excluded according to the exclusion criteria. The entire study adhered to data protection principles, and patient identity information was anonymized to ensure privacy and security.

### 2.6. Statistical power analysis

A post hoc statistical power analysis was performed for the primary outcome (SF-36 total score) using the observed between-group difference at 6 months and its standard deviation. Based on previous literature, the minimum clinically important difference (MCID) for the SF-36 total score in CR populations is approximately 5 points. In our study, the between-group difference at 6 months was 10.4 points (95% CI: 8.1–12.7), which exceeds the MCID threshold. Using these values, the calculated statistical power (2-tailed, α = 0.05) exceeded 0.90, indicating that the sample size was sufficient to detect clinically meaningful differences in the primary outcome.

### 2.7. Statistical analysis

All data were analyzed using SPSS 26.0 software (International Business Machines Corporation [IBM], Armonk ). For continuous variables that met the normal distribution assumption, data were presented as mean ± standard deviation ($\bar{x}$ ± s), and between-group comparisons were made using independent sample *t* tests. For non-normally distributed data, the median (interquartile range) was presented, and the Mann–Whitney *U* test was used for comparison. Categorical variables were presented as frequency and percentage, and differences between groups were assessed using the *χ*² test or Fisher exact probability method (when expected frequency < 5). To compare the changes in quality of life and other outcome measures between the intervention and control groups at different time points, repeated measures analysis of variance was used to assess the main effects of group, time, and their interaction. For variables that did not meet the assumptions of normality or homogeneity of variance, nonparametric methods were used. Baseline differences before and after matching were compared using the above methods, with PSM applied to control for confounding factors such as age, gender, and comorbidities using 1:1 nearest-neighbor matching. All statistical tests were 2-sided, with a *P* value < .05 indicating statistical significance. For missing data, multiple imputation was performed using a fully conditional specification method, generating 5 imputed datasets. Analyses were conducted on each dataset, and results were pooled according to Rubin rules. Sensitivity analysis was performed by comparing the results of the imputed dataset with those from a complete-case analysis, showing no material differences in the conclusions.

## 3. Results

### 3.1. Baseline characteristics comparison after PSM

After PSM, the rehabilitation program group (n = 62) and the conventional group (n = 62) were well balanced in terms of demographic characteristics, clinical features, and treatment plans (all *P* > .05). The 2 groups had similar average ages (71.6 ± 6.4 years vs 72.1 ± 6.1 years), with comparable male proportions (61.3% vs 64.5%). The distribution of major comorbidities (hypertension and diabetes) and revascularization methods (approximately 50% received PCI) was also balanced. Notably, the use of statins in both groups was close to 100%, and the use of β-blockers exceeded 95% (Table 1).

**Table 1:** Comparison of baseline clinical characteristics between the 2 groups of patients.

Variable	Rehabilitation group (n = 62)	Usual care group (n = 62)	*P*-value
Age (yr, mean ± SD)	71.6 ± 6.4	72.1 ± 6.1	.58
Male, n (%)	38 (61.3%)	40 (64.5%)	.712
Body mass index (kg/m²)	24.8 ± 3.2	25.1 ± 3.0	.562
Education level (high school or above)	35 (56.5%)	33 (53.2%)	.712
Smoking history, n (%)	29 (46.8%)	31 (50.0%)	.722
Hypertension, n (%)	45 (72.6%)	47 (75.8%)	.681
Diabetes mellitus, n (%)	28 (45.2%)	27 (43.5%)	.845
Hyperlipidemia, n (%)	36 (58.1%)	37 (59.7%)	.856
History of myocardial infarction, n (%)	22 (35.5%)	23 (37.1%)	.852
Underwent PCI, n (%)	31 (50.0%)	30 (48.4%)	.861
Underwent CABG, n (%)	5 (8.1%)	6 (9.7%)	.748
NYHA class ≥ III, n (%)	18 (29.0%)	20 (32.3%)	.698
Length of hospital stay (days)	8.7 ± 2.4	9.0 ± 2.1	.463
β-Blocker use, n (%)	60 (96.8%)	59 (95.2%)	.643
ACEI/ARB use, n (%)	54 (87.1%)	52 (83.9%)	.598
Statin use, n (%)	62 (100%)	61 (98.4%)	.316

CABG = coronary artery bypass grafting, PCI = percutaneous coronary intervention.

### 3.2. Changes in quality of life scores

Repeated measures analysis of variance revealed that the nurse-led CR program had a significant impact on improving patients’ quality of life. As shown in Table 2, the SF-36 total score demonstrated a significant group × time interaction effect at the 6-month follow-up (*P* = .023). The total score in the rehabilitation program group increased from baseline 58.2 ± 7.4 to 75.6 ± 5.8, which was significantly higher than the conventional group’s increase from 57.6 ± 6.9 to 65.2 ± 6.5 (between-group difference of 10.4 points, 95% CI 8.1–12.7).

**Table 2:** Changes in SF-36 scores over time between the 2 groups.

SF-36 domain	Time point	Rehabilitation group	Usual care group	Group effect (*P*)	Time effect (*P*)	Interaction (*P*)
Total score	Discharge	58.2 ± 7.4	57.6 ± 6.9	.281	<.001	.023
	3 months	69.5 ± 6.1	62.4 ± 7.0	.019		
	6 months	75.6 ± 5.8	65.2 ± 6.5	.011		
Physical functioning (PF)	Discharge	61.4 ± 8.1	60.7 ± 7.9	.311	<.001	.017
	3 months	72.1 ± 6.8	65.8 ± 7.4	.024		
	6 months	78.9 ± 6.2	68.5 ± 6.9	.008		
Role-physical (RP)	Discharge	54.3 ± 9.2	53.1 ± 9.5	.364	<.001	.032
	3 months	66.7 ± 8.5	58.9 ± 9.1	.036		
	6 months	72.4 ± 8.0	60.3 ± 8.8	.017		
General health (GH)	Discharge	60.5 ± 6.7	59.8 ± 6.5	.293	<.001	.022
	3 months	69.2 ± 5.9	63.3 ± 6.4	.028		
	6 months	74.6 ± 5.4	66.1 ± 6.2	.013		
Mental health (MH)	Discharge	59.5 ± 6.9	59.2 ± 7.1	.426	<.001	.045
	3 months	68.1 ± 5.6	63.4 ± 6.2	.031		
	6 months	73.3 ± 5.2	65.9 ± 6.0	.015		
Bodily pain (BP)	Discharge	57.0 (50.0, 64.0)	56.0 (49.0, 63.0)	.398	<.001	.029
	3 months	66.0 (60.0, 72.0)	60.0 (54.0, 67.0)	.044		
	6 months	71.0 (65.0, 77.0)	63.0 (56.0, 69.0)	.026		
Vitality (VT)	Discharge	54.0 (48.0, 60.0)	53.0 (47.0, 59.0)	.427	<.001	.033
	3 months	63.0 (57.0, 69.0)	58.0 (52.0, 64.0)	.041		
	6 months	69.0 (63.0, 74.0)	61.0 (55.0, 67.0)	.022		
Social functioning (SF)	Discharge	57.0 (50.0, 64.0)	56.0 (50.0, 63.0)	.295	<.001	.026
	3 months	67.0 (60.0, 73.0)	61.0 (55.0, 67.0)	.035		
	6 months	74.0 (67.0, 79.0)	64.0 (58.0, 70.0)	.018		
Role-emotional (RE)	Discharge	58.0 (51.0, 66.0)	57.0 (50.0, 64.0)	.338	<.001	.036
	3 months	68.0 (61.0, 75.0)	61.0 (54.0, 68.0)	.039		
	6 months	73.0 (66.0, 79.0)	63.0 (56.0, 70.0)	.02		

Among the dimensions, PF and SF showed the most significant improvements (interaction *P*-values of .017 and .026, respectively). At 6 months, the PF score in the rehabilitation group (78.9 ± 6.2) was 10.4 points higher than that of the conventional group (68.5 ± 6.9), and the median difference in SF scores reached 10 points (74.0 vs 64.0). Notably, subjective dimensions such as bodily pain (BP) and vitality (VT) also showed clinically meaningful improvements (median increase of 13–15 points), and all dimensions exhibited significant time effects (*P* < .001), indicating that both groups made progress over time, but the rehabilitation group showed significantly greater improvement.

### 3.3. Improvement in psychological status

The psychological assessment results showed that the nurse-led CR program had a significant impact on improving patients’ anxiety and depression symptoms (Table 3).

**Table 3:** Comparison of anxiety and depression symptom improvement between groups.

Outcome	Time point	Rehabilitation group	Usual care group	Interaction (*P*)
GAD-7 score	Discharge	7 [5–9]	7 [5–10]	.614
	3 months	4 [3–6]	6 [4–8]	.032
	6 months	3 [2–5]	6 [4–7]	.018
PHQ-9 score	Discharge	8 [6–10]	8 [6–11]	.572
	3 months	5 [3–7]	7 [5–9]	.027
	6 months	4 [2–6]	7 [5–8]	.011
≥3-point improvement in GAD-7	6 months	42 (67.7%)	28 (45.2%)	.015
≥3-point improvement in PHQ-9	6 months	39 (62.9%)	26 (41.9%)	.021

GAD = Generalized Anxiety Disorder, PHQ = Patient Health Questionnaire.

Regarding anxiety symptoms (GAD-7 scores), both groups had similar baseline levels at discharge [median score of 7 for both groups]. However, at 3 months, the rehabilitation group significantly reduced their GAD-7 score to 4 (IQR 3–6), while the conventional group remained at 6 (IQR 4–8), with a statistically significant between-group difference (interaction *P* = .032). By the 6-month follow-up, the rehabilitation group further improved to a score of 3 (IQR 2–5), significantly better than the conventional group, which had a score of 6 (IQR 4–7) (*P* = .018).

For depression symptoms (PHQ-9 scores), a similar trend was observed. The rehabilitation group reduced their PHQ-9 score from a baseline of 8 (IQR 6–10) to 4 (IQR 2–6) at 6 months, while the conventional group only decreased from 8 (IQR 6–11) to 7 (IQR 5–8) (interaction *P* = .011). Clinical significance analysis showed that the proportion of patients in the rehabilitation group achieving a GAD-7 score improvement of ≥3 points was significantly higher than in the conventional group (67.7% vs 45.2%, *P* = .015), and the proportion with a PHQ-9 score improvement of ≥3 points was also significantly higher (62.9% vs 41.9%, *P* = .021).

### 3.4. Improvement in exercise endurance

The 6MWT results showed that the nurse-led CR program significantly improved patients’ exercise endurance (Table 4). At discharge, the baseline walking distances were similar between the 2 groups [rehabilitation group 320m (IQR 290–350) vs conventional group 315 m (IQR 280–345), *P* = .58]. Over time, the rehabilitation group showed a more significant improvement trend (interaction *P* = .025).

**Table 4:** Comparison of Six-Minute Walk Test (6MWT) distance between groups over time.

Time point	Rehabilitation group (median [IQR], m)	Usual care group (median [IQR], m)	Group effect (*P*)	Time effect (*P*)	Interaction (*P*)
Discharge	320 [290–350]	315 [280–345]	.58	<.001	.025
3 months	360 [330–390]	335 [305–365]	.03		
6 months	380 [350–410]	345 [315–375]	.015		

At the 3-month follow-up, the rehabilitation group’s walking distance had increased to 360 m (IQR 330–390), which was significantly higher than the conventional group’s 335 m (IQR 305–365) (between-group comparison *P* = .03). By the 6-month follow-up, the rehabilitation group further improved to 380 m (IQR 350–410), while the conventional group only reached 345 m (IQR 315–375) (between-group comparison *P* = .015).

### 3.5. Improvement in cardiovascular risk factors

Blood pressure and lipid monitoring results indicated that the nurse-led CR program had a significant effect on controlling cardiovascular risk factors (Table 5). Regarding blood pressure control, the rehabilitation group showed a significant reduction in systolic blood pressure from baseline 135.4 ± 12.3 mm Hg to 125.3 ± 9.8 mm Hg at 6 months (*P* < .001), with a greater reduction compared to the conventional group (136.1 ± 13.1 mm Hg to 130.6 ± 10.9 mm Hg; interaction *P* = .038). Lipid profile improvement was particularly notable. The rehabilitation group’s low-density lipoprotein cholesterol level decreased to 90.5 ± 22.9 mg/dL at 6 months, which was 14.8 mg/dL lower than the conventional group (105.3 ± 25.2 mg/dL, *P* = .012). Additionally, high-density lipoprotein cholesterol levels significantly increased in the rehabilitation group (52.4 ± 11.3 mg/dL vs 47.3 ± 9.7 mg/dL, *P* = .019). Triglyceride levels also showed more significant improvement in the rehabilitation group (120.7 ± 48.3 mg/dL vs 138.5 ± 52.1 mg/dL, *P* = .027).

**Table 5:** Comparison of cardiovascular risk factor improvements between groups.

Time point	Rehabilitation group (mean ± SD)	Usual care group (mean ± SD)	Group effect (*P*)	Time effect (*P*)	Interaction (*P*)
Systolic blood pressure (mm Hg)					
Discharge	135.4 ± 12.3	136.1 ± 13.1	.742	<.001	.038
3 months	128.7 ± 10.5	132.4 ± 11.7	.045		
6 months	125.3 ± 9.8	130.6 ± 10.9	.021		
Diastolic blood pressure (mm Hg)					
Discharge	78.5 ± 8.2	79.0 ± 7.9	.823	<.001	.045
3 months	74.2 ± 7.1	77.0 ± 7.6	.053		
6 months	72.1 ± 6.8	75.8 ± 7.2	.026		
LDL-C (mg/dL)					
Discharge	115.6 ± 28.3	116.4 ± 29.1	.882	<.001	.041
3 months	98.2 ± 24.7	108.7 ± 26.5	.034		
6 months	90.5 ± 22.9	105.3 ± 25.2	.012		
HDL-C (mg/dL)					
Discharge	45.3 ± 10.1	44.9 ± 9.8	.796	.002	.057
3 months	49.7 ± 10.7	46.1 ± 9.5	.048		
6 months	52.4 ± 11.3	47.3 ± 9.7	.019		
Triglycerides (mg/dL)					
Discharge	145.2 ± 55.6	148.9 ± 53.2	.768	.015	.049
3 months	130.4 ± 50.2	140.7 ± 51.8	.081		
6 months	120.7 ± 48.3	138.5 ± 52.1	.027		

HDL-C = high-density lipoprotein cholesterol, LDL-C = low-density lipoprotein cholesterol.

### 3.6. Comparison of health behavior adherence and clinical outcomes

The nurse-led CR program significantly improved patients’ health behavior adherence and reduced readmission risk (Table 6). Regarding dietary adherence, the rehabilitation group showed a significantly higher adherence rate at 3 months (77.4%, 48/62) compared to the conventional group (56.5%, 35/62) (*P* = .012). This advantage further increased at 6 months (82.3% vs 53.2%, *P* = .005), with a significant group × time interaction effect (*P* = .045). More notably, the rehabilitation group had a 6-month readmission rate of only 9.7% (6/62), which was significantly lower than the conventional group’s 22.6% (14/62) (*P* = .048).

**Table 6:** Comparison of health behavior adherence and clinical outcomes between groups.

Time point	Rehabilitation group (n, %)	Usual care group (n, %)	Group effect (*P*)	Time effect (*P*)	Interaction (*P*)
Dietary adherence rate					
3 months	48 (77.4%)	35 (56.5%)	.012	.008	.045
6 months	51 (82.3%)	33 (53.2%)	.005		
Rehospitalization rate					
6 months	6 (9.7%)	14 (22.6%)	.048	–	–

## 4. Discussion

In this study, we systematically evaluated the long-term effects of a nurse-led CR program on quality of life, psychological status, and functional status in elderly patients with CAD. The results showed that, after 6 months of follow-up, patients who received nurse-led rehabilitation management demonstrated significant advantages over those who received conventional discharge guidance across multiple core outcome measures, further supporting the value of this model for clinical practice.

First, regarding overall quality of life improvement, the rehabilitation group showed a significant increase in SF-36 total scores at the 6-month follow-up, which was significantly better than the conventional group. The improvement was most notable in the PF and SF dimensions, suggesting that nurse-led interventions not only enhanced patients’ physical recovery but also promoted their social engagement abilities. This improvement may stem from the goal-setting and continuous remote interventions initiated by nursing staff before discharge, which allowed patients to have a higher degree of awareness and involvement in the rehabilitation process, thus improving adherence and proactivity in treatment.^[13,14]^ Compared to the physician-led model, which typically emphasizes exercise rehabilitation, the nurse-led rehabilitation pathway places more focus on behavioral interventions and personalized support, making it more feasible and acceptable for elderly populations. Consistent with Riegel research on the role of “continuity of care” in managing chronic diseases, our intervention effectively delayed the decline of health behaviors through frequent follow-ups and platform interactions.^[12,15]^

Second, the improvement in psychological status was also notably significant. At the 6-month follow-up, patients in the rehabilitation group showed a marked reduction in GAD-7 and PHQ-9 scores, with greater relief from anxiety and depression symptoms compared to the conventional group. This outcome may be attributed to the role of nurses as “emotion regulators” during rehabilitation.^[16]^ Elderly CAD patients often experience psychological distress due to disease uncertainty, functional limitations, and anxiety about the future, and nurses, with their higher frequency of contact and willingness to communicate, can identify and intervene in negative emotions early. Compared to traditional medication-focused approaches for managing anxiety/depression, the emotional support, experience sharing, and group support provided by nurse-led interventions offer a more flexible and empathetic psychological intervention channel for elderly patients.^[17]^ This finding aligns with studies from abroad, which have highlighted the role of nurses in emotional regulation in the management of chronic diseases in elderly populations, and reflects the current advocacy for a “person-centered” approach to chronic disease care.

The superior outcomes observed in the nurse-led rehabilitation group may be partly explained by Bandura self-efficacy theory, which posits that an individual’s confidence in their ability to perform a specific behavior strongly influences the likelihood of adopting and maintaining that behavior. In our intervention, nurses provided individualized goal setting, frequent feedback through digital platforms, and positive reinforcement, which collectively enhanced patients’ self-efficacy in managing exercise, diet, and medication adherence. Moreover, the model aligns with the principles of the continuity of care theory, emphasizing sustained, coordinated support across the transition from hospital to home. Continuous follow-up and emotional support from familiar nursing staff likely mitigated psychological distress, reinforced health-related behaviors, and maintained patient engagement over time. These mechanisms suggest that the combination of personalized care, ongoing monitoring, and emotional connection inherent in nurse-led models creates a more favorable environment for long-term behavioral change and improved clinical outcomes compared to conventional, less continuous approaches.

Finally, in terms of functional status, the rehabilitation group showed a far greater improvement in 6MWT compared to the conventional group, reflecting the effectiveness of nurse-led rehabilitation in promoting the recovery of patients’ aerobic capacity and physical activity levels. Unlike traditional models, nurse-led rehabilitation programs focus more on monitoring daily living behaviors and providing goal feedback. The information platform captures changes in activity levels in real-time and provides encouragement and guidance. This continuous feedback mechanism may be one of the key factors driving functional recovery.^[18]^ Previous studies have pointed out that patients often experience adherence issues in physician-driven exercise programs, whereas nurses, with their more practical and life-relevant guidance combined with graphical feedback to reinforce intrinsic motivation, are better suited to guiding the elderly, who are typically more dependent.

Furthermore, the results of this study indicate that the nurse-led rehabilitation pathway has a strong time effect, meaning that the intervention’s impact continues to enhance over time. This finding suggests that continuous care and periodic assessments are key to maximizing rehabilitation outcomes. In contrast to previous criticisms of “short-term interventions with unsustainable long-term effects,” the nurse-led rehabilitation model relies on regular follow-ups and dynamic adjustment mechanisms, ensuring that patients remain in a state of continuous attention and support. This helps reduce the risk of behavior discontinuity and relapse.^[19,20]^ This approach aligns with the concept of “maintenance phase management” proposed in current nursing intervention research.

In this study, the rehabilitation group demonstrated a mean improvement of +65.2 m in the 6MWT from baseline to 6 months, exceeding the MCID of 30 to 50 m reported by Perera et al,^[21]^ suggesting that the observed functional improvement is not only statistically significant but also clinically meaningful. Similarly, the SF-36 total score increased by 10.4 points, surpassing the MCID threshold of 5 to 10 points described by Ware et al,^[22]^ indicating a substantial and clinically relevant enhancement in quality of life. These findings further support the practical value of the nurse-led CR program in elderly CAD patients.

While this study preliminarily confirms the positive impact of the nurse-led CR program on improving the quality of life in elderly CAD patients, several limitations remain. First, as a single-center retrospective cohort study, this research has certain limitations regarding sample representativeness and causal inference ability. Although propensity score matching was used to control for confounding factors, there may still be unknown or unmeasurable biases, such as patient willingness to engage in rehabilitation and social psychological variables like family support, which were not included in the analysis. Secondly, the success of the rehabilitation intervention depends on patient adherence and their ability to use the platform; some elderly patients may face difficulties with remote operations and online interactions, which could affect the actual effectiveness of the intervention. Additionally, the 6-month follow-up period allowed for an initial assessment of the short-to-medium term effects of the rehabilitation plan, but the long-term impact on quality of life and cardiovascular event prognosis still requires further study. Lastly, quality of life and psychological state evaluations mainly relied on subjective scales, which may be subject to response biases. Future research could incorporate more objective physiological and behavioral data for supplementary validation. Therefore, future studies may consider adopting a multicenter, prospective randomized controlled design with extended follow-up periods and integration of multimodal data to strengthen the generalizability and scientific rigor of the evidence.

Overall, the findings of this study not only reaffirm the important role of CR in improving the quality of life of elderly CAD patients but also highlight the unique value of the nurse-led model in improving rehabilitation participation rates, optimizing intervention pathways, alleviating psychological burdens, and enhancing quality of life. This model, through the integration of health education, emotional support, functional training, and remote technologies, forms a closed-loop rehabilitation chain covering both the hospitalization and community phases, better meeting the “continuous, personalized, and multi-dimensional” rehabilitation needs of elderly individuals. Future research could further explore the heterogeneity of responses to nurse-led rehabilitation in different subgroups (e.g., those with diabetes, low educational levels, or living alone) and the mediating role of nurse–patient interaction frequency and platform usage habits on outcomes. Additionally, conducting prospective research based on multicenter or community-based studies would provide more robust evidence to support the widespread implementation of nurse-led rehabilitation.

## Author contributions

**Conceptualization:** Juan Feng, Shasha Li, Jie Liu.

**Data curation:** Juan Feng, Shasha Li, Jie Liu.

**Formal analysis:** Juan Feng, Shasha Li, Jie Liu.

**Investigation:** Juan Feng, Shasha Li, Jie Liu.

**Methodology:** Juan Feng, Shasha Li, Jie Liu.

**Supervision:** Juan Feng, Shasha Li, Jie Liu.

**Validation:** Shasha Li, Jie Liu.

**Visualization:** Juan Feng, Shasha Li, Jie Liu.

**Writing – original draft:** Juan Feng, Jie Liu.

**Writing – review & editing:** Juan Feng, Jie Liu.
